# AdipoRon Protects against Tubular Injury in Diabetic Nephropathy by Inhibiting Endoplasmic Reticulum Stress

**DOI:** 10.1155/2020/6104375

**Published:** 2020-08-06

**Authors:** Shan Xiong, Yachun Han, Peng Gao, Hao Zhao, Na Jiang, Lin Sun

**Affiliations:** Department of Nephrology, The Second Xiangya Hospital, Central South University, Changsha, Hunan 410011, China

## Abstract

Endoplasmic reticulum (ER) stress has been reported to play a pivotal role in diabetic nephropathy (DN). AdipoRon is a newly developed adiponectin receptor agonist that provides beneficial effects for diabetic mice; however, its underlying mechanism remains to be delineated. Here, we demonstrated increased expression levels of ER stress markers, accompanied by upregulated levels of proinflammatory cytokines and increased expression of collagen I, fibronectin, Bax, and cleaved caspase 3 in the kidneys of db/db mice compared with control mice. Decreased expression of adiponectin receptor 1 (AdipoR1) and phosphorylated 5′AMP-activated kinase (p-AMPK) was also observed in the kidneys of db/db mice. However, these alterations were partially reversed by intragastric gavage with AdipoRon. *In vitro*, AdipoRon alleviated high-glucose-induced ER stress, oxidative stress, and apoptosis in HK-2 cells, a human tubular cell line. Moreover, AdipoRon restored the expression of AdipoR1 and p-AMPK in HK-2 cells exposed to high-glucose conditions. Additionally, these effects were partially abrogated by pretreatment with AdipoR1 siRNA, but this abrogation was ameliorated by cotreatment with AICAR, an AMPK activator. Furthermore, the effects of AdipoRon were also partially abolished by cotreatment with compound C. Together, these results suggest that AdipoRon exerts favorable effects on diabetes-induced tubular injury in DN by inhibiting ER stress mediated by the AdipoR1/p-AMPK pathway.

## 1. Introduction

Diabetic nephropathy (DN) is the primary cause of end-stage renal disease for which better therapies are urgently needed [1]. Well-described characteristic pathophysiological features of DN include glomerular mesangial expansion and hypertrophy, tubular hypertrophy, and extracellular matrix deposition in the glomerular and tubulointerstitial compartments [2]. Although the pathogenesis of DN is still unclear, accumulating evidence has shown that endoplasmic reticulum (ER) stress plays a vital role in its onset and progression.

The ER serves as a central site for folding, posttranslational modification, and transport of cellular proteins. Disruption of cellular homeostasis leads to the accumulation of misfolded and unfolded proteins, activating a signaling pathway referred to as the unfolded protein response (UPR). In unstressed conditions, the three ER sensors, including protein kinase-like ER kinase (PERK), inositol requiring protein-1*α* (IRE1*α*), and activating transcription factor-6 (ATF6), are inactive and are bound by glucose-regulated protein of 78 kDa (GRP78), an ER chaperone protein. During ER stress, GRP78 dissociates from the three sensors and facilitates their activation and their associated signaling pathways [3]. However, sustained and prolonged induction of ER stress tends to be cytotoxic, for instance, leading to apoptosis [4, 5].

Recently, the pivotal role of ER stress in DN progression, especially in tubulointerstitial injury, has been well-documented [6]. Moreover, abundant studies have indicated that suppression of ER stress protects against diabetic kidney injury [7, 8]. Furthermore, the expression of UPR-associated genes, including GRP78 and X-box binding protein 1 (XBP1), was upregulated in renal biopsies from patients with DN [7]. Of note, several studies indicated that TUDCA and 4-PBA, classic ER stress inhibitors, could alleviate diabetic kidney damage in a diabetic murine model [9, 10]. In addition, erlotinib, an epidermal growth factor receptor inhibitor, was also found to inhibit the progression of DN by attenuating ER stress [11]. These findings suggest that inhibition of ER stress may be a feasible therapeutic strategy for treating DN. Nevertheless, novel agents targeting ER stress need to be further investigated.

Adiponectin is an adipokine secreted by adipocytes that improves insulin sensitivity, mitigates the inflammatory response, and inhibits fibrosis [12]. Recent studies have indicated that plasma adiponectin levels are reduced in obese and type 2 diabetic patients [13], and adiponectin administration improved insulin resistance in diabetic mice [14, 15]. However, there are serious adverse effects of having excess adiponectin [16]. AdipoRon is a newly developed adiponectin receptor agonist, which could exert similar positive effects of adiponectin but deliver few side effects caused by adiponectin overexpression. It has been reported that AdipoRon improved insulin sensitivity and expanded life span in db/db mice by activating the adiponectin receptor 1- (AdipoR1-) phosphorylated 5′AMP-activated kinase (p-AMPK) pathway and the adiponectin receptor 2- (AdipoR2-) peroxisome proliferative-activated receptor-*α* (PPAR*α*) pathway, respectively [17]. In addition, a recent study revealed that AdipoRon administration ameliorates diabetes-induced glomerular endothelial cell and podocyte injury in db/db mice by activating the intracellular CaMKK*β* (Ca^2+^/calmodulin-dependent protein kinase kinase-*β*)/LKB1 (liver kinase B1)-AMPK/PPAR*α* pathway [18], suggesting an effective therapeutic strategy for treating DN. Nonetheless, the impact and underlying mechanism of AdipoRon in diabetic nephropathy, particularly tubular injury, remain largely unknown. Given the findings that activation of AMPK attenuates ER stress, apoptosis, and kidney fibrosis [19, 20], we speculate that AdipoRon might exert protective effects on diabetic tubular injury via inhibition of ER stress mediated by the AdipoR1/AMPK pathway. Therefore, our study investigated whether ER stress inhibition is involved in the renoprotective effect of AdipoRon in db/db mice and in human proximal tubular epithelial cell lines.

## 2. Methods and Materials

### 2.1. Antibodies and Reagents

The following commercial antibodies were used in our study. Anti-ADIPOR1 (ab126611), anti-AMPK*α*1 (ab32047), anti-collagen I (ab34710), and anti-fibronectin (ab2413) were obtained from Abcam. Anti-phospho-PERK (Thr980) (3179), anti-phospho-AMPK*α* (Thr172) (2535), anti-CHOP (2895S), anti-GRP78 (3177S), and anti-cleaved caspase 3 (9661S) were purchased from Cell Signaling Technology. Anti-PERK (AF5304) and anti-Bcl-2 (AF6139) were from Affinity. Anti-CHOP (GB34710) was from Servicebio. Anti-BAX (60267-1-Ig) and anti-*β*-actin (60008-1-Ig) were obtained from Proteintech. All secondary antibodies used for western blot analysis were purchased from Abcam. AdipoRon was from SELLECK. AICAR, compound C, and TUDCA (S3654) were also purchased from SELLECK.

### 2.2. Animal Experimental Design

Fourteen-week-old male C57BLKS/J db/m mice and C57BLKS/J db/db mice used for animal experiments were obtained from the Aier Matt Experimental Animal Company (China). These mice were randomly divided into three groups: db/m mice (control, *n* = 8), db/db mice (vehicle control, *n* = 8), and db/db mice receiving AdipoRon via intragastric gavage (*n* = 8). AdipoRon (30 mg/kg) was dissolved in 0.5% sodium carboxymethyl cellulose solution and was provided to db/db mice once daily via intragastric gavage from 16 weeks of age for 4 weeks. The blood glucose (sampled from the tail) and body weight were measured twice a week. At 20 weeks of age, all mice were euthanized. Kidneys and blood samples were collected for further analysis. All animal procedures were carried out following the protocol approved by The Animal Care and Use Committee of Second Xiangya Hospital of Central South University.

### 2.3. Assessment of Physiologic Features and Renal Functions

Body weight and tail blood glucose were measured and collected twice a week. Twenty-four-hour urine sample collections were performed using metabolic cages. Urine albumin concentrations and serum creatinine were tested using an Albuwell M kit and a creatinine assay kit (Exocell Inc.) in accordance with the manufacturer's instructions, respectively.

### 2.4. Morphological Analysis of Renal Tissues

Renal tissue samples were fixed in 4% paraformaldehyde for 24 hours, dehydrated, and embedded in paraffin. Four-micrometer-thick paraffin-embedded renal tissue sections from three mouse groups were prepared and then subjected to periodic acid-Schiff (PAS) staining, hematoxylin and eosin (HE) staining, and Masson's trichrome staining. Tubular damage was analyzed using the Tervaet semiquantitative scoring system as previously described [21].

### 2.5. Immunofluorescence (IF)

Four-micrometer-thick formalin-fixed, paraffin-embedded renal tissue sections from three mouse groups were prepared for IF studies. After deparaffinization and hydration, the sections were placed in a container and covered with 1 mM EDTA (pH 8.0) at 95°C for 20 minutes for antigen retrieval. The sections were then blocked with 5% bovine serum albumin for 30 minutes and incubated overnight at 4°C with primary antibodies against AdipoR1 (1 : 100 dilution), GRP78 (1 : 100 dilution), or CHOP (1 : 100 dilution). After washing three times with PBS, the slides were incubated with secondary antibodies in the dark at room temperature for 50 minutes. The sections were then counterstained with DAPI in the dark for 10 minutes. Images were collected using confocal microscopy and analyzed using ImageJ.

### 2.6. Detection of Superoxide Generation

Four-*μ*m-thick cryostat tissue sections were prepared and stained with dihydroethidium (DHE, Sigma-Aldrich) to detect intracellular reactive oxygen species (ROS) production in the kidney tubules. 2′,7′-Dichlorodihydrofluorescein diacetate (H2-DCFDA, Invitrogen) was used to monitor intracellular ROS production in experimental HK-2 cells according to the manufacturer's instructions. Both treated tissue sections and cells were examined by confocal microscopy and then were analyzed by calculating the mean fluorescence intensity of 10-20 randomly selected fields.

### 2.7. TUNEL Staining

TUNEL staining was carried out to assess apoptotic cells in renal tissue sections from three mouse groups using an In Situ Cell Death Detection Kit (Roche Life Science) according to the manufacturer's instructions.

### 2.8. Quantitative Reverse-Transcriptase PCR (qRT-PCR)

Total RNA extraction of renal tissues or experimental HK-2 cells was performed using a TRIzol reagent (TaKaRa). Reverse transcription was then carried out to generate complementary DNA using TaqMan RT reagents (TaKaRa) following the protocol provided by the manufacturer. Quantitative real-time PCR was subsequently performed using a TB GreenTM Premix Ex Taq II reagent (TaKaRa) as previously described. The detected mRNA levels were normalized by gene actin-beta (ACTB). All reactions were performed in triplicate.

The sequences of the mouse primers are listed as follows. ACTB: forward 5′-TAGCCATCCAGGCTGTGCTG-3′, reverse 5′-CAGGATCTTCATGAGGTAGTC-3′; MCP-1: forward 5′-TAAAAACCTGGATCGGAACCAAA-3′, reverse 5′-GCATTAGCTTCAG ATTTACGGGT-3′; IL-6: forward 5′-ACTTCCATCCAGTTGCCTTCTTGG-3′, reverse 5′-TTAAGCCTCCGACTTGTGAAGTGG-3′; and TNF*α*: forward 5′-GCGACGTGGAACTGGCAGAAG-3′, reverse 5′-GCCACAAGCAGGAATGAGAAGAGG-3′.

The sequences of the human primers are listed as follows. ACTB: fwd 5′-CATGTACGTTGCTATCCAGGC-3′, rev 5′-CTCCTTAATGTCACGCACGAT-3′; MCP-1: forward 5′-CAGCCAGATGCAATCAATGCC-3′, reverse 5′-TGGAATCCTGAACCCACTTCT-3′; IL-6: forward 5′-ACTCACCTCTTCAGAACGAATTG-3′, reverse 5′-CCATCTTTGGAAGGTTCAGGTTG-3′; and TNF*α*: forward 5′-CCTCTCTCTAATCAGCCCTCTG-3′, reverse 5′-GAGGACCTGGGAGTAGATGAG-3′.

### 2.9. Cell Culture and Treatments

Human proximal tubular epithelial cell lines (HK-2) obtained from ATCC were used in our *in vitro* studies. HK-2 cells were cultured in media containing different concentrations of D-glucose (5.6 or 30 mM), with or without AdipoRon (5, 10, or 50 *μ*mol/L), AICAR (0.5 mM), or compound C (20 *μ*M). Furthermore, for gene disruption, cells were pretreated with AdipoR1 siRNA using a Lipofectamine 300 reagent (Invitrogen) according to the manufacturer's instructions.

### 2.10. Western Blot (WB) Analysis

Total proteins of renal tissues or treated HK-2 cells were extracted using ice-cold RIPA buffer (CWBIO) supplemented with phosphatase inhibitors and protease inhibitors (CWBIO). Thereafter, the protein concentration was determined with a BCA assay. Total proteins were then subjected to 6%-12% SDS-PAGE and transferred onto nitrocellulose membranes. After blocking with 5% bovine serum albumin (BSA), membranes were incubated with multiple primary antibodies, including anti-*β*-actin (1 : 5000), anti-AdipoR1 (1 : 2000), anti-AMPK*α*1 (1 : 3000), anti-phospho-AMPK*α* (1 : 1000), anti-collagen I (1 : 5000), anti-fibronectin (1 : 5000), anti-phospho-PERK (1 : 1000), anti-PERK (1 : 1000), anti-CHOP (1 : 1000), anti-GRP78 (1 : 1000), anti-cleaved caspase 3 (1 : 1000), anti-Bcl-2 (1 : 1500), and anti-BAX (1 : 3000). After overnight incubation at 4°C, membranes were then incubated with horseradish peroxidase-conjugated secondary antibodies (Abcam) at room temperature for 40-60 minutes. The membrane blots were detected using an enhanced chemiluminescence kit (Thermo Scientific). Bands were quantified by ImageJ as previously described [22].

### 2.11. Cell Immunofluorescence

Treated HK-2 cells were fixed with 4% paraformaldehyde, permeabilized, and blocked with 5% BSA for 60 minutes at room temperature. After incubating with antibodies directly against AdipoR1 (1 : 100 dilution) overnight, cells were washed with PBS and reincubated with secondary antibodies conjugated with Alexa Fluor (Abcam) for 60 minutes at 37°C. Cells were counterstained with Hoechst and were visualized by an LSM 780 META laser scanning microscope.

### 2.12. Statistical Analysis

The experimental data were presented as the mean ± standard deviation and were compared statistically by performing one-way analysis of variance (ANOVA). *P* < 0.05 was considered statistically significant.

## 3. Results

### 3.1. AdipoRon Improves Renal Functional and Morphological Changes in db/db Mice

**Figure 1:**
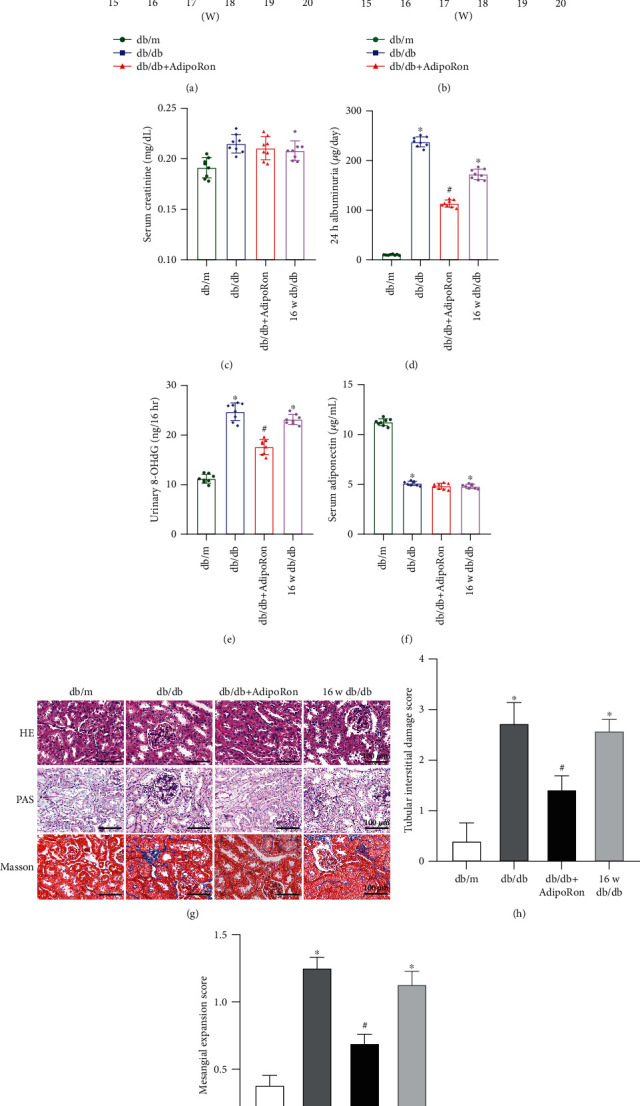
Effects of AdipoRon on renal functional and morphological changes in db/db mice: (a) body weight changes in three mouse groups from 16 weeks to 20 weeks; (b) blood glucose levels; (c) serum creatinine concentrations; (d) proteinuria/24 h in each group; (e) urinary 8-OHdG levels; (f) serum adiponectin levels; (g) HE (upper panels), PAS (middle panels), and Masson staining (bottom panels) of renal tissue sections from db/m, db/db, db/db mice treated with AdipoRon, and 16-week-old db/db mice before the treatment. *n* = 8 mice per group. Bars = 100 *μ*m. (h, i) Quantitative analysis of the tubular interstitial damage score and mesangial expansion score in each group. The data are expressed as the means ± SDs. ^∗^*P* < 0.05 versus db/m control mice; ^#^*P* < 0.05 versus db/db mice. HE: hematoxylin and eosin; PAS: periodic acid-Schiff.

Compared with nondiabetic db/m mice, db/db mice exhibited higher body weight and blood glucose levels (Figures 1(a) and 1(b)). AdipoRon treatment slightly improved blood glucose levels in db/db mice, whereas it had no effect on body weight (Figures 1(a) and 1(b)). Moreover, there were no significant differences in serum creatinine in the four mouse groups (Figure 1(c)). Obvious decreases in albuminuria and in urinary oxidative stress marker 8-OHdG levels were observed in db/db mice treated with AdipoRon compared to those of db/db control mice and diabetic mice before the treatment (Figures 1(d) and 1(e)). In addition, AdipoRon treatment had no effect on serum adiponectin levels (Figure 1(f)). HE and PAS staining showed notable glomerular mesangial expansion and tubular interstitial damage in diabetic db/db mice and diabetic mice before the treatment compared with db/m mice, and these changes were attenuated by AdipoRon treatment (Figures 1(g)–1(i)). Moreover, Masson staining showed mildly increased interstitial fibrosis in db/db mice and those before the treatment compared with db/m mice, while this increase was reversed by AdipoRon treatment (Figure 1(g)). These ameliorations in renal morphological parameters and renal function in db/db mice by AdipoRon treatment to the degree somewhat comparable to those of nondiabetic db/m mice indicate its reversal effect in DN progression.

### 3.2. AdipoRon Diminishes Inflammation, Apoptosis, and Fibrosis in the Kidneys of db/db Mice

**Figure 2:**
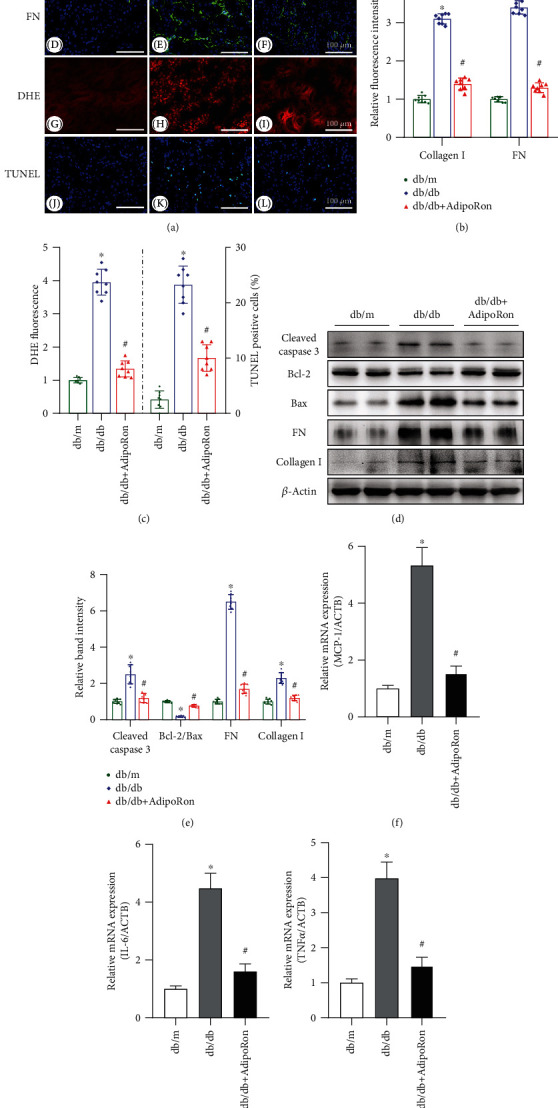
Effects of AdipoRon on inflammation, apoptosis, and fibrosis in the kidneys of db/db mice. (a) Representative immunofluorescence images of collagen I (A–C) and FN (D–F) expression in the renal tissues of db/m, db/db, and db/db mice treated with AdipoRon. Oxidative stress and apoptosis in renal tissues were detected using DHE reagents (G–I) and TUNEL staining (J–L), respectively. *n* = 8 mice per group. Bars = 100 *μ*m. (b) Semiquantitative analysis of the protein levels of collagen I and FN detected by IF. (c) Semiquantitative assessment of oxidative stress and apoptosis in each group. (d) Western blot analysis of cleaved caspase 3, Bcl-2, Bax, FN, and collagen I. *β*-Actin was used as a loading control. *n* = 8 mice per group. (e) Relative band density of cleaved caspase 3, Bcl-2/Bax, FN, and collagen I expression. (f–h) mRNA levels of MCP-1, IL-6, and TNF*α* in the renal tissues of each group determined by qRT-PCR. The data are shown as the mean ± SD. ^∗^*P* < 0.05 versus db/m control; ^#^*P* < 0.05 versus db/db mice. FN: fibronectin; DHE: dihydroethidium; TUNEL: terminal deoxynucleotidyl transferase-mediated dUTP nick end labeling; MCP-1: monocyte chemotactic protein-1; IL-6: interleukin-6; TNF*α*: tumor necrosis factor *α*; qRT-PCR: quantitative reverse-transcriptase PCR.

As shown in Figures 2(a) and 2(b), the levels of collagen I and fibronectin (FN) were notably increased in diabetic db/db mice compared with control db/m mice, as assessed by immunofluorescence staining; however, these changes were partially reversed by AdipoRon treatment. Similar results were further obtained from western blot assays (Figures 2(d) and 2(e)). In addition, DHE and TUNEL staining revealed significant increases in oxidative stress and tubular epithelial apoptosis in the kidneys of db/db mice compared to those of control mice, but all of these alterations were significantly reversed by AdipoRon treatment (Figures 2(a) and 2(c)). Moreover, western blot analysis showed that the ratio of Bcl-2/Bax was decreased and the protein expression of cleaved caspase 3 was upregulated in db/db mice but partially restored after AdipoRon treatment (Figures 2(d) and 2(e)). The expression of proinflammatory cytokines, such as interleukin-6 (IL-6), monocyte chemoattractant protein-1 (MCP-1), and tumor necrosis factor-*α* (TNF*α*), was notably increased in the kidney tissues of db/db mice as detected by quantitative reverse-transcriptase polymerase chain reaction (qRT-PCR), and these changes were reversed by AdipoRon treatment (Figures 2(f)–2(h)). These results suggested that AdipoRon can inhibit the expression of proinflammatory cytokines, apoptosis, and fibrosis in the kidneys of diabetic mice.

### 3.3. AdipoRon Restores the Expression of AdipoR1 and the Phosphorylation Level of AMPK along with Reducing the Expression of GRP78, CHOP, and p-PERK in the Kidneys of db/db Mice

**Figure 3:**
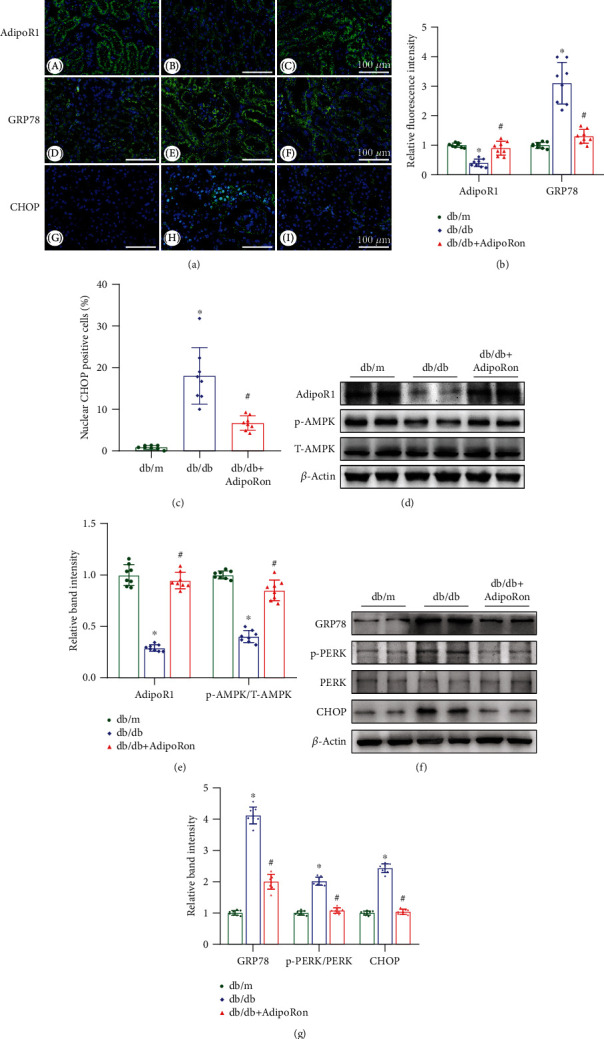
Role of AdipoRon in the expression of AdipoR1 and p-AMPK and ER stress in the kidneys of db/db mice. (a) Representative immunofluorescence images of AdipoR1 (A–C), GRP78 (D–F), and CHOP (G–I) in the renal tissues of three mouse groups. *n* = 8 per group. Bars = 100 *μ*m. (b) Semiquantitative analysis of AdipoR1 and p-AMPK expression. (c) Semiquantitative analysis of nuclear CHOP positive cells (%). (d) Representative immunoblots of AdipoR1, p-AMPK, and T-AMPK. *β*-Actin was used as a loading control. (e) Relative band intensity. (f) Representative immunoblots of GRP78, p-PERK, PERK, and CHOP. (g) Relative band intensity. *n* = 8 mice per group. The data are shown as the means ± SDs. ^∗^*P* < 0.05 versus db/m control mice; ^#^*P* < 0.05 versus db/db mice. AdipoR1: adiponectin receptor 1; GRP78: glucose-regulated protein of 78 kDa; CHOP: C/EBP homologous protein; p-AMPK: phosphorylated 5′AMP-activated kinase; PERK: protein kinase-like endoplasmic reticulum kinase; p-PERK: phospho-PERK.

Immunofluorescence staining revealed markedly lower adiponectin receptor 1 (AdipoR1) expression in the kidneys of db/db mice compared with those of db/m controls, while the change was reversed by AdipoRon treatment (Figures 3(a) and 3(b)). Moreover, consistent with the decrease of AdipoR1, phosphor-Thr^172^ AMPK, a principle downstream signal in the AdipoR1 pathway, was notably decreased in db/db mice but partially restored following AdipoRon treatment (Figures 3(d) and 3(e)). To evaluate the effect of AdipoRon on ER stress, the expression of ER stress markers, including GRP78 and CHOP, was examined. As shown in Figures 3(a)–3(c), the expression levels of GRP78 and CHOP were increased in the kidneys of db/db mice but decreased after AdipoRon treatment. These findings were further confirmed by immunoblot analyses (Figures 3(f) and 3(g)). As detected by western blot analysis, the level of phosphorylated PERK was elevated in the kidneys of db/db mice compared with those of db/m mice, whereas this change was reversed by AdipoRon treatment (Figures 3(f) and 3(g)). Taken together, these results suggested that ER stress was induced in the kidneys of db/db mice and could be inhibited by treatment with AdipoRon.

### 3.4. AdipoRon Attenuates the Upregulated Expression of ER Stress Markers, Proinflammatory Cytokines, Apoptosis, and Fibrosis in HK-2 Cells Exposed to High-Glucose Conditions

**Figure 4:**
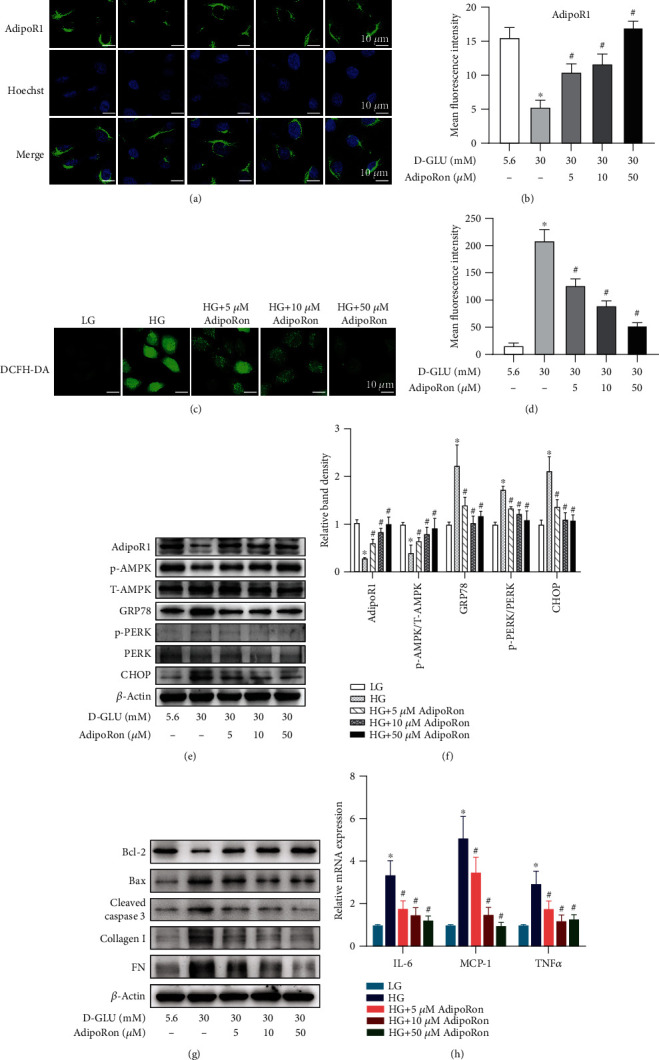
Effects of AdipoRon treatment on ER stress, inflammation, apoptosis, and fibrosis in HK-2 cells under HG conditions. (a) Representative confocal images of AdipoR1 in HK-2 cells treated or not treated with different concentrations of AdipoRon (5 *μ*M, 10 *μ*M, and 50 *μ*M) under HG conditions. *n* = 3 per group. Bars = 10 *μ*m. (b) Quantification of the average immunofluorescence intensity of AdipoR1 in HK-2 cells. (c) The levels of intracellular ROS in treated HK-2 cells as assessed by DCFH-DA staining. *n* = 3 per group. Bars = 10 *μ*m. (d) Bars graphs depict intracellular ROS content in treated HK-2 cells. (e) Representative western blot bands of AdipoR1, p-AMPK, T-AMPK, GRP78, p-PERK, PERK, and CHOP in HK-2 cells treated with different concentrations of AdipoRon under HG conditions. *β*-Actin was used as a loading control. *n* = 3 per group. (f) Relative band density of WB. (g) Immunoblot assays of Bcl-2, Bax, cleaved caspase 3, collagen I, and FN in treated HK-2 cells. *n* = 3 per group. (h) mRNA levels of MCP-1, IL-6, and TNF*α* in HK-2 cells treated or not treated with different concentrations of AdipoRon (5 *μ*M, 10 *μ*M, and 50 *μ*M) under HG conditions. *n* = 3 per group. The data are shown as the means ± SDs. ^∗^*P* < 0.05 versus LG; ^#^*P* < 0.05 versus HG. LG: low glucose; HG: high glucose; ROS: reactive oxygen species; *μ*M: *μ*mol/L.

To investigate the effects of AdipoRon in vitro, human proximal tubular epithelial cell lines (HK-2) cultured in high-glucose (HG) medium (30 mmol/L D-glucose) were treated with different concentrations of AdipoRon. As indicated in Figures 4(a) and 4(b), AdipoRon effectively increased the expression of AdipoR1 in a dose-dependent manner, which was accompanied by activation of AMPK (Figures 4(e) and 4(f)). Consistent with the in vivo findings, AdipoRon inhibited HG-induced ER stress, as determined by decreased GRP78, p-PERK, and CHOP expression in HK-2 cells (Figures 4(e) and 4(f)). Moreover, AdipoRon significantly attenuated HG-induced elevated production of intracellular reactive oxygen species as evidenced by DCFH-DA staining (Figures 4(c) and 4(d)). Furthermore, the mRNA expression of proinflammatory cytokines, such as IL-6, MCP-1, and TNF*α*, was increased significantly in HK-2 cells exposed to HG ambience compared to the control, while the increases were blocked partially by AdipoRon treatment (Figure 4(h)). In addition, decreased expression of Bcl-2 and elevated expression of Bax and cleaved caspase 3 were noted in HK-2 cells with HG stimulation, whereas these alterations were reversed by AdipoRon treatment (Figure 4(g)). Furthermore, western blot analysis showed that the expression of FN and collagen I was notably increased in HK-2 cells exposed to HG ambience but partially reduced in those treated with AdipoRon (Figure 4(g)).

### 3.5. AdipoRon Inhibits HG-Induced ER Stress in Part via the AdipoR1/p-AMPK Pathway

**Figure 5:**
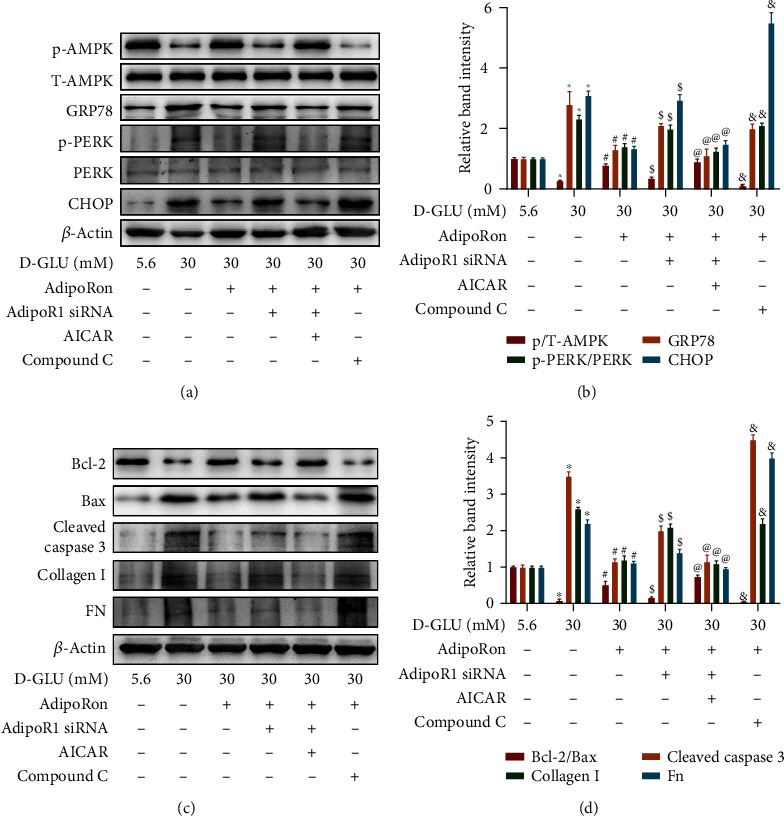
Effect of AdipoR1 siRNA, AICAR, and compound C on the levels of p-AMPK and ER stress in HK-2 cells treated with AdipoRon under HG conditions. (a) Immunoblot assays of the protein expression of p-AMPK, T-AMPK, GRP78, p-PERK, PERK, and CHOP in HK-2 cells treated with AdipoRon, AdipoR1 siRNA, AICAR, or compound C under HG ambience. *n* = 3 per group. (b) Relative band density. (c) Representative western blot bands of Bcl-2, Bax, cleaved caspase 3, collagen I, and FN in HK-2 cells treated with AdipoRon, AdipoR1 siRNA, AICAR, or compound C under HG conditions. *n* = 3 per group. (d) Relative band density. The data are shown as the means ± SDs. ^∗^*P* < 0.05 compared to the LG group; ^#^*P* < 0.05 compared to the HG group. ^$^*P* compared to the HG+AdipoRon group. ^@^*P* < 0.05 compared to the HG+AdipoRon+AdipoR1 siRNA group. ^&^*P* compared to the HG+AdipoRon group. AICAR: 5-amino-1-[(2R,3R,4S,5R)-3,4-dihydroxy-5-(hydroxymethyl)oxolan-2-yl]imidazole-4-carboxamide.

To determine whether the protective effect of AdipoRon on ER stress is modulated by the AdipoR1/p-AMPK pathway, the AdipoR1 small interfering RNA (siRNA), the AMPK activator AICAR, and the AMPK inhibitor compound C were used. Immunoblot analyses revealed that the HG-induced suppression of AMPK phosphorylation in HK-2 cells was reversed by AdipoRon treatment, and this effect was partially abrogated by transfection with the AdipoR1 siRNA. However, the effect of AdipoR1 siRNA was partially abolished by cotreatment with AICAR, an AMPK agonist. In addition, the effect of AdipoRon on the increased level of p-AMPK in HK-2 cells was also blocked by cotreatment with compound C (Figures 5(a) and 5(b)). Conversely, AdipoRon inhibited HG-induced ER stress in HK-2 cells, as indicated by decreased levels of GRP78, p-PERK, and CHOP expression. These effects, however, were partially blocked by cotreatment with AdipoR1 siRNA or compound C. Meanwhile, cotreatment with AICAR reversed the effect of AdipoR1 siRNA transfection on ER stress (Figures 5(a) and 5(b)). Moreover, similar results were observed for the expression of Bax and cleaved caspase 3, as well as the levels of collagen I and FN (Figures 5(c) and 5(d)). These data suggest that AdipoRon regulates HG-induced ER stress and fibrosis in part through the AdipoR1/p-AMPK pathway.

## 4. Discussion

Recently, diabetic tubular injury has become a hot topic of investigation, as it has been recognized to be a better indicator of DN progression than glomerular pathology [23]. Disruption of cellular ER homeostasis in the kidney contributes largely to tubular damage, thereby leading to the development and progression of DN. Therefore, appropriate regulation of renal ER stress seems to be a promising therapeutic approach for treating DN. In this study, we observed that AdipoRon restored the expression of AdipoR1 in DN, along with an apparent increase in AMPK phosphorylation, thereby attenuating diabetes-induced ER stress, inflammation, apoptosis, and fibrosis in the tubules of DN. Our study revealed for the first time that AdipoRon treatment can ameliorate diabetes-induced tubular injury by regulating ER stress and that this effect occurs partially through the AdipoR1/p-AMPK pathway.

Of note, AdipoRon has been recently reported to ameliorate diabetic kidney injury in db/db mice [18]. Likewise, our study revealed that AdipoRon improved renal function and attenuated proteinuria, oxidative stress, and renal pathology injury in type 2 diabetic db/db mice (Figure 1). Moreover, we also found that AdipoRon alleviated intrarenal inflammation, apoptosis, and fibrosis in the kidneys of db/db mice (Figure 2), suggesting that AdipoRon may be a promising strategy for treating DN.

To investigate the mechanism underlying the therapeutic effects of AdipoRon on DN, we focused our attention on ER stress. Previous studies have shown that ER stress is related to the pathogenesis of diabetic tubulointerstitial injury [7, 8]. Furthermore, notable ER stress has been detected in the tubulointerstitial compartment of patients with DN [7, 24], suggesting its vital role in the development and progression of DN. In this study, increased ER stress in the tubules was also observed in db/db diabetic mice, as demonstrated by increased levels of the ER stress markers, such as GRP78 and CHOP (Figure 3). Notably, 4-PBA and TUDCA, chemical ER chaperones that can enhance the folding capacity of ER, were found to decrease albuminuria, alleviate mesangial expansion, and inhibit apoptosis in a diabetic mouse model via inhibition of ER stress [9, 10]. Moreover, some herbal or plant medicines, such as astragaloside IV and oleanolic acid, were also shown to alleviate diabetic kidney damage by suppressing ER stress [25, 26]. These results prompted us to speculate that normalization of ER stress by therapeutic strategies might attenuate tubular injury, thereby slowing or reversing the progression of DN. Therefore, the identification of novel drugs or approaches with respect to regulating renal ER stress requires further study.

Although research on the inhibition of ER stress by AdipoRon is somewhat limited, a few studies have reported that adiponectin could attenuate ER stress in chronic intermittent hypoxia-induced kidney injury [27]. Since AdipoRon is a novel oral adiponectin receptor agonist, which can exert similar positive effects as adiponectin, and since AdipoR1 is predominantly expressed in renal proximal tubules, we speculate that AdipoRon may alleviate tubular injury in DN by inhibiting ER stress in tubular cells. As expected, AdipoRon attenuated tubular injury in the kidneys of db/db mice (Figure 1), and there was an accompanying reduction in ER stress (Figure 3). These findings suggested that inhibition of ER stress is involved in the beneficial effects of AdipoRon toward diabetic tubular damage.

Inflammation is also a pivotal player in the development of DN. Moreover, ER stress has been reported to promote the activation of proinflammatory transcription factors, such as NF-*κ*B [28], and to promote the expression of MCP-1 in diabetic kidneys [29]. Here, our results also confirmed the inhibitory effects of AdipoRon on inflammation in DN. As shown in Figure 2, AdipoRon decreased diabetes-induced transcription of proinflammatory cytokines, such as IL-6, MCP-1, and TNF*α* in the kidney of db/db mice. Moreover, AdipoRon also reduced the production of renal ROS in the tubules of db/db mice (Figure 2).

In addition, apoptosis has also been shown to be closely linked with ER stress [4]. The transcription factor CHOP, also termed DDIT3, is a mediator of apoptosis. Persistent ER stress leads to upregulated expression of CHOP, which then promotes the transcription of Bim, a BH3-only protein of the BCL-2 family that is required for apoptosis, and downregulates the expression of BCL-2, ultimately resulting in the induction of apoptosis [30]. In contrast, loss of CHOP prevented tunicamycin-induced apoptosis in kidney epithelial cells [31]. Therefore, we also verified the role of AdipoRon in apoptosis in diabetic db/db mice. As expected, AdipoRon decreased TUNEL staining, suppressed caspase 3 activation, and restored the ratio of Bcl-2/Bax in the kidneys of db/db mice (Figure 2).

Renal fibrosis is a major pathogenic feature of DN and is closely related to the progression of renal dysfunction [32]. It is commonly believed that ER stress, inflammation, and apoptosis are associated with fibrosis. Therefore, we also examined the effects of AdipoRon on fibrosis in db/db mice. Expectedly, increased expression of collagen I and fibronectin was observed in the kidneys of db/db mice compared to those of the controls, whereas the elevation of these factors was apparently inhibited by treatment with AdipoRon (Figure 2).

The next question needs to be addressed of the underlying mechanism by which AdipoRon attenuated ER stress. As reported, AdipoRon may exert its anti-inflammatory and antioxidant effects via two adiponectin receptors and their associated pathways [17]. In addition, AdipoR1 promotes the activation of the AMPK pathway, whereas AdipoR2 is linked with PPAR*α* activation. Of note, AMPK is a critical regulator of cellular energy balance and metabolism in multiple cells. Activation of AMPK plays a crucial role in defensive response to stress. As reported, AMPK activation protects against kidney injury in chronic kidney disease [33–35]. Furthermore, AMPK has been shown to improve vascular function via inhibition of ER stress [35]. Interestingly, activation of AMPK via metformin suppressed tunicamycin-induced ER stress in HK-2 cells and inhibited renal fibrosis in a unilateral ureteral obstruction mouse model [20]. These findings raise the possibility that activation of AMPK might improve diabetic kidney injury by attenuating ER stress. In this regard, we hypothesized that AdipoRon protects against diabetes-induced ER stress possibly through the AdipoR1/p-AMPK signaling pathway.

First, we detected the levels of AdipoR1 and p-AMPK in mouse kidneys. As expected, AdipoRon increased the expression of AdipoR1 and p-AMPK in the kidneys of db/db mice compared with those of control mice (Figure 3). Consistent with the in vivo study, similar results were obtained in HK-2 cells exposed to HG conditions (Figure 4). Interestingly, higher doses (50 *μ*M) of AdipoRon rather than lower doses (5 *μ*M or 10 *μ*M) seemed to be more effective in restoring the expression of AdipoR1 in HK-2 cells exposed to HG conditions (Figure 4); thus, we chose 50 *μ*M AdipoRon for additional mechanism studies. Our results indicated that the genetic suppression of AdipoR1 via siRNA in HK-2 cells abrogated the effects of AdipoRon on HG-induced ER stress (Figure 5), whereas this abrogation was partially reversed by pharmacological activation of AMPK with AICAR. In contrast, pharmacological inhibition of AMPK in HK-2 cells by treatment with compound C abolished the effects of AdipoRon on HG-induced ER stress and was accompanied by increased expression of Bax, cleaved caspase 3, collagen I, and fibronectin (Figure 5). Overall, these findings suggest that the AdipoR1/p-AMPK pathway might participate in the favorable effects of AdipoRon on DN.

Nevertheless, despite our abundant investigatory efforts, there are still some concerns remaining to be solved in our study. For instance, although the expression of AdipoR2 has been shown to be relatively scarce in renal tissues [36, 37], recent reports by Park et al. [18, 38] showed that the renal expression of AdipoR2 was somewhat comparable to the level of AdipoR1 and was necessary for AdipoRon to exert favorable lipid metabolic effects in diabetic mice. Here, our study mainly focused attention on the role of AdipoR1 in the inhibition of ER stress induced by AdipoRon treatment in tubular cells, but whether and how the activation of AdipoR2 is involved in the amelioration of ER stress remain unknown. Notwithstanding that little research so far has been done to investigate the association between AdipoR2 and ER stress in DN, a study by Liu et al. reported that the activation of PPAR*α* (the primary downstream effector of AdipoR2) by adiponectin alleviated ER stress and apoptosis through transcriptional inhibition of ATF2 in mouse adipose [39]. Combined with these findings, we speculate that the activation of AdipoR2 by AdipoRon treatment may also account for the amelioration of ER stress in DN, which will be investigated in our future study. Additionally, the mechanism by which AMPK regulates ER stress in db/db mice treated with AdipoRon deserves to be further determined. Inspired by previous findings that activation of AMPK by metformin inhibited ER stress through increasing the expression of hemeoxygenase-1 in tubular cells [20] and suppressing NADPH oxidase-derived ROS in endothelial cells [40], we will go on exploring in our future study.

## 5. Conclusions

In conclusion, our work demonstrated the novel therapeutic effects of AdipoRon on tubular injury in DN using in vivo and in vitro models. The underlying mechanism of these effects might involve modulating ER stress, ameliorating inflammation, and inhibiting apoptosis and fibrosis. Moreover, these effects tend to be mediated by activating intrarenal AdipoR1, which in turn promotes the activation of the AMPK pathway. Taken together, these findings suggest that the adiponectin receptor agonist AdipoRon is a promising therapeutic agent for treating DN.

## Data Availability

The data used to support the findings of this study are included within the article.

## Abbreviations

AdipoR1: Adiponectin receptor 1
AdipoR2: Adiponectin receptor 2
AMPK: Adenosine 5′-monophosphate-activated protein kinase
AICAR: 5-Amino-1-[(2R,3R,4S,5R)-3,4-dihydroxy-5-(hydroxymethyl)oxolan-2-yl]imidazole-4-carboxamide
ATF6: Activating transcription factor-6
BSA: Bovine serum albumin
CHOP: C/EBP homologous protein
DHE: Dihydroethidium
DN: Diabetic nephropathy
ER: Endoplasmic reticulum
FN: Fibronectin
GRP78: Glucose-regulated protein of 78 kDa
HE: Hematoxylin and eosin
HG: High glucose
H2-DCFDA: 2′,7′-Dichlorodihydrofluorescein diacetate
IRE1*α*: Inositol requiring protein-1*α*
IL-6: Interleukin-6
IF: Immunofluorescence
LG: Low glucose
MCP-1: Monocyte chemotactic protein-1
PAS: Periodic acid-Schiff
PERK: Protein kinase-like endoplasmic reticulum kinase
PPAR*α*: Peroxisome proliferative-activated receptor-*α*
qRT-PCR: Quantitative reverse-transcriptase polymerase chain reaction
siRNAs: Small interfering RNAs
TUNEL: Terminal deoxynucleotidyl transferase-mediated dUTP nick end labeling
TNF*α*: Tumor necrosis factor *α*
UPR: Unfolded protein response
WB: Western blot
XBP1: X-box binding protein 1.
